# Graphical Presentations in Systemic Anticancer Treatment Network Meta-Analyses: A Systematic Review

**DOI:** 10.3233/SHTI231071

**Published:** 2024-01-25

**Authors:** Sandeep JAIN, Xuanyi LI, Jeremy WARNER

**Affiliations:** a Warren Alpert Medical School of Brown University, Providence, RI, USA; b Vanderbilt University Medical Center, Nashville, TN, USA; c Lifespan Cancer Institute, Providence, RI, USA

**Keywords:** Data visualization, cancer, network meta-analysis, network graph

## Abstract

Network meta-analysis (NMA) draws conclusions about indirect comparisons of randomized clinical trials and is considered high-level evidence. Most NMA publications make use of network plots to portray results. Network plots are complex graphics that can have many visual attributes to portray useful information, such as node size, color, and graph layout. We analyzed the network plots from 162 NMAs of systemic anticancer therapy efficacy using a set of 16 attributes. Our review showed that the current state of network plot data visualizations within the NMA space lacks diversity and does not make use of many of the visual attributes available to convey information. More thoughtful design choices should be placed behind these important visualizations, which can carry clinical significance and help derive treatment plans for patients.

## 1. Introduction

The treatment of cancer depends on systemic anticancer therapies (SACT). New systemic therapies, or combinations of therapies known as regimens, are adopted into practice if they show outcomes that are better or comparable to previous standards of care. These comparisons are often made through randomized clinical trials (RCTs), with a standard control arm comparator usually required for regulatory approval. RCTs are incredibly expensive and time-consuming and are often sponsored by the maker of a particular pharmaceutical agent. As such, it is impractical and virtually impossible to perform head-to-head trials between all SACT for any given cancer type.

One alternative to performing a prospective RCT is using existing data from previous RCTs to make indirect drug-to-drug comparisons across a common denominator, a technique known as network meta-analysis (NMA) [1]. Rigorously performed NMA are considered high-level evidence and may influence clinical practice.

One of the key methods for conveying NMA results is through a network plot to visually portray the direct and indirect comparisons of the included studies. Nodes represent therapies, and edges represent a *direct* comparison between the two therapies at either end. Network plots sometimes make use of node and edge attributes (e.g., color, node size, edge width, overall layout) to encode information about the therapies and their respective relationships in the network. For instance, the width of an edge connecting two nodes may represent the number of trials that have compared the two therapies, or the number of patients studied. These plot attributes are likely to influence a viewer’s perception of the data and ultimately may have implications on patient care.

Making conclusions based on graph attributes is a complex cognitive task that has been rigorously studied. In 1984, Cleveland and McGill studied how humans decode information in the human-graph interface through ten different elementary perceptual tasks [2]. A human’s ability to perceive data is attribute dependent. For example, their study revealed that we are more accurate at perceiving differences in length compared to differences in area. This difference in perception emphasizes the importance of attribute selection. How data is represented in an NMA plot may have implications on the conclusions that are made by the readers. To our knowledge, a systemic review of NMA graphs has not been conducted. Here, we aim to thoroughly describe these attributes in the domain of SACT efficacy. We chose this domain due to its relative complexity, increasing prevalence of NMA literature, and homogeneity.

## 2. Methods

On October 14, 2022, we searched MEDLINE using the search string “*neoplasms[mesh] + Network Meta-Analysis[mesh]*”. This search yielded 680 unique articles. Article titles were screened to determine if the articles involved: 1) NMA; 2) SACT. Of the 680 titles, 209 were deemed relevant based on the title alone; 1 additional article [3] was added despite failing the title screening, based on prior knowledge. Of these 210, 18 were not available for review. Of the remaining 192 articles, 28 did not contain network plots in any portion of the article, including supplementary materials. On further review, 2 of the remaining 164 articles were related to SACT toxicity, not efficacy, and were eliminated from our repository. The remaining 162 articles were used for our attribute descriptions.

We described the articles’ network plots using a data dictionary that contained 2 integer fields: # of network plots and # of nodes in each plot; 1 categorical: layout of the network plot (circle, force-based, radial, mesh, custom, unknown); and 13 binaries: presence of labels on or near nodes; if node labels, necessity of a node label legend/key; presence of labels on or near the edges; if edge labels, necessity of an edge label legend/key; discernable contrast between the edge and node color; presence of directionality; presence of a non-white background; and variation in node size; node color; node shape; edge color; edge style; and edge width. These variables were determined *a priori* based on visualization theory.

To verify data dictionary reliability and usability, we required two separate reviewers have at least a 90% inter-annotator agreement when describing the selected articles using the 16 data dictionary fields. 10 articles from our repository were randomly selected for review. A total of 160 elements were compared (16 features across 10 randomly selected articles). There was disagreement on 10 elements out of a total of 160 (93.75% agreement). Disagreements were resolved through consensus discussion after which full data extraction proceeded.

## 3. Results

Table 1 summarizes the characteristics of the articles that were analyzed in our study. Thirteen different cancer groupings were represented. The most frequent cancer group was gastrointestinal malignancy (41 NMAs) followed by thoracic cancer (36 NMAs). Articles were published across a broad array of 82 separate journals, with a generally increasing trend from n=6 in 2016 to n=35 in 2022. The most common journal featured was *Critical Reviews in Oncology/Hematology* (n=10; 2022 impact factor [IF]=6.312) followed by *Oncotarget* (n=9; 2022 IF=3.331). Of the 162 articles reviewed, 74 (46%) contained a single network plot. The remaining 88 articles contained anywhere from 2 to 13 separate network plots. Of note, when an article contained more than one network plot, the overall style and formatting across the individual plots within the article were identical in all but 5 articles. In one article, the distinct network plot conveyed toxicity information rather than efficacy and was excluded from our review. The remaining four articles had 2 distinct styles with unique attributes. Each article-style dyad was reviewed separately. In other words, across 162 distinct articles, we reviewed 166 article-style dyads. Overall, a total of 441 network plots were observed. The median number of nodes per network plot was 8.

The remaining article-style dyad attributes are shown in Table 2. The most common layout was the circle layout. Most plots did not make use of node color, node shape, edge color, or edge labels. Node size and edge width were common features that were used to convey data. In 20 (12%) of the article-style dyads, the network plots used the same color or grayscale intensity for the nodes as the edges.

## 4. Discussion

SACT NMA serves as a tool to help summarize evidence in a large variety of cancer types and will likely increase in importance given the limitations of creating head-to-head prospective RCTs. The statistical challenges of NMA have been reviewed thoroughly in the literature [4]; however, little-to-no emphasis has been placed on the visualizations of the network plots, despite the potential utility. The network plots used within NMAs are important in understanding the comparisons that are being made across therapies and can help viewers comprehend complex data; conversely an uninformative plot can conceal information or even lead to erroneous conclusions. Making decisions based off these network plots depends on the human-graph interface, and this interface is determined by the attributes used in the plots.

Many visual channels were underutilized based on our review. Although the reasons for this are unclear, we speculate that most of the reports used the default plot settings of the most popular Windows-based NMA software, WinBUGS. Each visual attribute has potential in conveying important and distinct information about NMA if used to its full potential. For example, the most used plot layout throughout the articles reviewed was the circular layout, which is adequate for small graphs but quickly leads to a “hairball” effect once node and edge count are moderately large. Other layouts, such as a force-based approaches, can portray network centrality and place greater significance on the therapies that were most directly compared. The use of node colors, largely left out of the articles we reviewed, could be used to portray therapeutic modalities, and help viewers realize when therapies with similar or dissimilar mechanisms of action were being compared.

**Figure 1.**
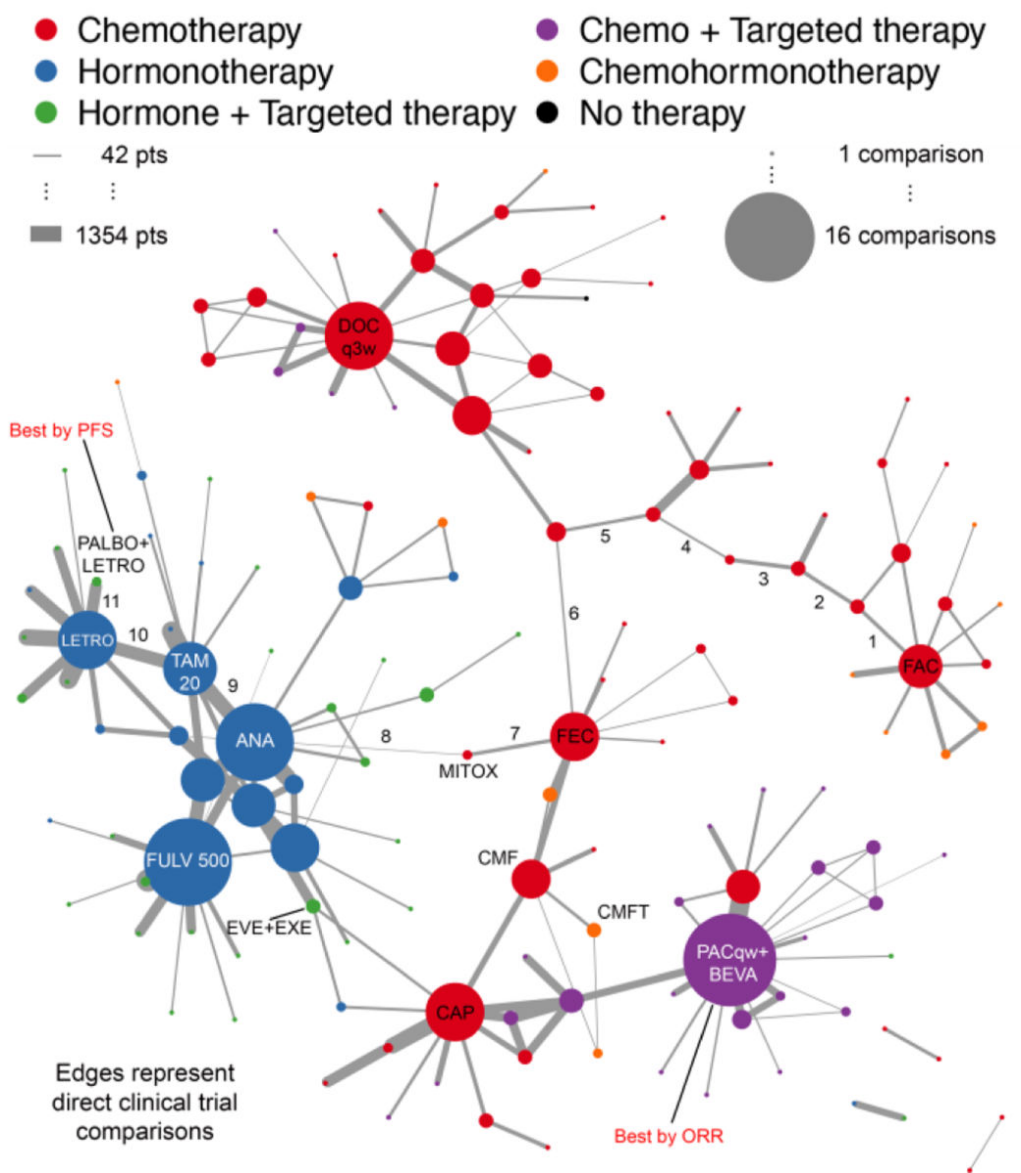
A reconstructed network plot using the same set of RCTs from Giuliano et al. The numeric labels represent the minimum path from FAC to PALBO+LETRO, demonstrating a highly indirect comparison which is completely obscured in a standard circular layout.

To demonstrate the potential of visual attributes, we have reconstructed plots from Giuliano et al., a very large NMA of metastatic breast cancer therapies with 2 separate network graphs, containing 95 and 112 nodes, respectively [2,5]. The original plots had circular layout, many overlapping edges, and a single node color. This reconstruction (Figure 1) makes use of several attributes, including node color, edge width, force-based layout, and node size; only some nodes are labeled to reduce visual clutter. Use of colors helps portray how SACT comparisons are clustered by mechanism. We numbered edges to portray the highly indirect nature of some of the comparisons in the NMA. Without these attributes, these insights would remain hidden to the reader.

## 5. Conclusions

Network plots play an integral role in understanding NMA.

Although we focused on SACT efficacy NMA in order to limit the heterogeneity of the studies, our findings are likely applicable to toxicity NMA and to NMA in other biomedical domains, especially those that have a large number of treatment options. Without proper design choices, network plots are a missed opportunity at best and potentially misleading at worst. NMA researchers and publishers should reconsider the dominant use of circular layouts and other uninformative plot attributes and more formal data visualization research should be conducted into optimal network plot design.

## Figures and Tables

**Table 1.** Summary of the cancer types, publications, year of publication and number of graphs contained within our repository of oncology systemic therapy network meta-analysis articles.

Cancer Type	Journal of Publication	Year Published	# of Graphs per Article	# of Nodes
Gastrointestinal: 41	*Crit Rev Onc/Hem*: 10	2016: 6	Minimum: 1	Min: 3
Thoracic: 36	*Oncotarget*: 9	2017: 18	25^th^ %ile: 1	25^th^ %ile: 5
Genitourinary: 22	*BMC Cancer*: 8	2018: 20	Median: 2	Median: 8
Plasma Cell: 12	*Medicine (Baltimore)*: 6	2019: 20	75^th^ %ile: 4	75^th^ %ile: 11
Breast: 12	*PLoS One*: 5	2020: 27	Max: 13	Max: 112
Head & Neck: 10	*BMJ Open*: 4	2021: 36		
7 other cancers: 29	76 other journals: 120	2022: 35		

**Table 2.** Summary of 13 of the attributes that were collected across n=166 article-style dyads. T represents “true” and ‘F’ represents “false”.

Layout, N (%)	Node Color	Node Label	Node Label Encoded	Node Size	Node Shape	Edge Color	Edge Style
Circle: 127 (76)	T: 21 (13)	T: 166 (100)	T: 100 (60)	T: 96 (58)	T: 5 (3)	T: 23 (14)	T: 19 (11)
Custom: 23 (14)	F: 145 (87)	F: 0 (0)	F: 66 (40)	F: 70 (42)	F: 161 (97)	F: 143 (86)	F: 147 (89)
Unknown: 9 (5)							
Radial: 5 (3)	Edge Width	Edge Label	Edge Label Encoded		Edge Node Difference	Directionality	
Force: 1 (1)	T: 113 (68)	T: 57 (34)	T: 33 (58)		T: 146 (88)	T: 4 (2)	
Mesh: 1 (1)	F: 53 (32)	F: 109 (66)	F: 24 (42)		F: 20 (12)	F: 162 (98)	
